# Supplementary material for: Modification with Conventional Surfactants to Improve a Lipid-Based Ionic-Liquid-Associated Transcutaneous Anticancer Vaccine
Source: Molecules. 2023 Mar 27;28(7):2969. doi: 10.3390/molecules28072969 (PMC10095727; doi:10.3390/molecules28072969)
Supplement: Supplementary file 1 [file molecules-28-02969-s001.zip › molecules-2272123-supplementary.pdf]

molecules

Supplementary Materials

S-1. Supportive Methodology

S-1.1: Cellular Toxicity by MTT Assay Method

The LabCyte EPIMODEL cell-containing cup was incubated in 500 μL of assay medium for 24 h (at 37 °C with 5% CO_2). After incubation, the 50 μL tested sample was added to the cup and incubated for another 24 h. The cell-containing cup was washed (10–15 times) with PBS to remove all residue from the cup before adding 500 μL of 0.5 mg/mL MTT assay medium and incubating for 3 h. The cellular tissues were immersed in 500 μL of isopropyl alcohol (IPA) and stored at 4 °C in the dark for 48 h. One hundred μL of this extracted solution was placed in a 96-well ELISA plate, and the absorbance was measured using a micro-plate reader at 570 nm and 650 nm wavelengths to determine the number of viable cells. As a blank solution, IPA was used, and IPM or PBS was used as a negative control with 100% cell viability.

S-1.2: Quantitively Analysis of IMQ by HPLC System

The assay amount of imiquimod (IMQ) was quantified by the high-performance liquid chromatography (HPLC) system (JASCO International Co. Ltd., Tokyo, Japan). Photodiode array (PDA) detector MD-4010, inertsil ODS-3, 4.6 × 250 mm, 5 μm, C_{18} reverse phase column (GL Sciences Inc., Tokyo, Japan) was used with 1.0 mL/min flow of mobile phase. The mobile phase contained an equal volume ratio (50:50) of acetate buffer (pH 3.7) and acetonitrile. The retention time of IMQ was around 3.6 min at 244 nm (λ_{max}).

S-1.3: Skin Cryosection Study for TDDS

In vitro: CLSM (with 20× lenses) was used to assess the depth of FITC-OVA penetration into YMP-skin. In summary, 200 μL of the tested sample were applied to the skin with FDCS, the skin was unclasped from the Franz cell after 48 h, and the desired area was cut after washing with 20% ethanol. Soaked for 6 h in 0.4% formaldehyde solutions, they were then microtome-sliced into 20 μm thick slices and mounted on a glass slide with mounting solution. The depth of FITC-OVA penetration into the YMP-skin was measured using ImageJ software after visualizing it with CLSM.

In vivo: The depth of FITC-OVA penetration into the C57BL/6N mice skin was evaluated using the skin cryosection technique with a microtome and CLSM (with 20× lenses). In short, 100 μL of the examined sample was applied to the clean mouse's dorsal skin for 6 h using home-made patches. To remove the extra tested sample, the treated skin region was rinsed with a 20% ethanolic solution, then immersed in a 0.4% paraformaldehyde solution, and stored at −80 °C overnight. CLSM was utilized to observe drug penetration into the skin after the skin tissues were sliced into 20 μm thick slices with a microtome and placed on glass slides. The depth of FITC-OVA permeation into the mouse skin was calculated using ImageJ software.

S-1.4: Detection of IgG Specific Antibody by ELISA

Expression of the ovalbumin (OVA)-specific immunoglobulin-G (IgG) titer and it's subclass (IgG-1 and IgG-2a) were investigated by enzyme-linked immunosorbent assay (ELISA) techniques. In brief, the OVA- aqueous solutions (200 μL; 5.0 mg/mL OVA solution) were coated in 96-well polystyrene plates (Maxisorp, NUNC) by keeping them in the refrigerator for overnight. Wash the plate with PBST solution (0.1% Tween-20 in PBS), then added 200 μL of blocking buffer (2.0 wt.% BSA from Wako Pure Chemical Industries), and incubated for 2 h at room temperature, and washed again with PBST. Following then, 20 μL of diluted plasma serum (diluted at 1:50, 1:250, 1:1250, and 1:6250 with 1 wt.% BSA solution) was added to each well of 96- well ELISA plate and incubated at 37 °C for

2 h. Washed again with PBST, and 100 μL of anti-mouse antibody (diluted antibody labeled with rabbit anti-mouse IgG, IgG1, and IgG2a) was added. The cells were then incubated at 37 °C for 2 h. Washed the plate with PBST and added the 100 μL tetramethylbenzidine (TMB) solutions (Wako Pure Chemical Industries), then kept it for around 30 min at room temperature (dark place is preferable) to initiate the horseradish peroxidase (HRP) reaction. After that, the reaction was stopped by adding 0.1 N HCl solutions, and the color intensity of the solutions was measured by the microplate reader (iMARK, Bio-Rad, Tokyo, Japan) at 450 nm. Controlled or non-treated samples were diluted before immunization, and the concentration of antibody titers (IgG, IgG-1, and IgG-2a) was determined by calculating the inverse dilutions where the optical density of the serum samples was equal to the control sample.

S-1.5: Tumor Challenges

The antitumor effect of nanovaccine was observed by measuring the tumor volume (tumor growth and development), body weight variation, and survival rate of C57BL/6N mice. The tumor was budding after 7–9 days of subcutaneous inoculation of 100 μL (containing 2 × 10^6 cells) of the OVA specific tumor drive cell EG7-OVA in C57BL/6N mice. Body weight and tumor volume were assessed at 1-day intervals starting on the day the tumor became evident, using a weight measuring scale (balance machine) and slide calipers. The volume of the tumor was calculated using the equation below:

$$\text{Tumor volume [mm}^3\text{]} = (\text{length [mm]}) \times (\text{width[mm]})^2 \times 0.5. \quad (1)$$

The tumor volume was used to calculate the survival rate, and mice were considered clinically dead when the tumor volume exceeded 2500 mm^3. In addition, the body weight variance was calculated using the inoculating day as the starting point.

S-1.6: Flow Cytometric Analysis

One hundred μL of Cy5-OVA-sample solutions (containing 200 μg of OVA and 100 μg of IMQ) were applied to the C57BL/6N mouse skin's dorsal region for 24 h using TP and SI. After 24 h, the required applied skin-tissue and lymph node were harvested and cleaned with ethanol and PBS before being sliced into minute pieces and digested with 50 U/mL DNAse-I and 300 g/mL liberase before being incubated at 37 °C for 1.5 h.

The digested tissues were then filtered using a 70 μm cell strainer and prepared as a single cell suspension (1 × 10^6 cells/mL) in 1 mL of 2% fetal bovine serum (FBS) in PBS (FBS-PBS). The upper supernatant component of the cell suspension was withdrawn and re-added in 1 mL of FBS-PBS after centrifugation at 800 ×g for 6 min. The cell suspensions were incubated at 4 °C for 30 min with 100 μL of 1 μg/mL Alexa-488-labeled anti-CD207+ antibody and PE/Cy7-labeled anti-CD103+ antibody to determine the expression of Langerhans cells (LCs; CD207+ CD103-) and dendritic cells (DCs; CD207+ CD103+). Following that, the antibody specific cell expression was analyzed using a multi-channel flow cytometer (EC800, Sony, Japan). The expression of CD207 was used to distinguish skin DCs from other cell populations. LCs and dDCs were distinguished from the CD207+ cell population by CD103 expression. The antigen uptake ratios of these two cell groups were estimated using the formula below.

$$\text{Antigen uptake ratio [\%]} = [(\text{antigen+ LCs or dDCs}) / \{(\text{antigen+ LCs or dDCs}) + (\text{antigen- LCs or dDCs})\}] \times 100 \quad (2)$$

S-1.7: Tumor Microenvironment (TME) Observation

The cytotoxic CD8+ T-Cells' expression in the tumor microenvironment was observed by the tumor cryosection. In a brief, the tumor was grown in C57BL/6N mice by subcutaneously inoculating 100 μL (contained 2 × 10^6 cells) of OVA-specific tumor drive cell EG7-OVA. Tumor budding takes 7–10 days to develop, and mice were then

immunized with 100 μL of sample solutions containing 200 μg of OVA through TP and SI. The tumor was collected and bisected for cryosection 7 days after the third consecutive dosages were given at 7-day intervals. The desired tumor was rinsed in PBS solutions before being immersed in 0.4% formaldehyde solutions for 4 h. It was then frizzed at −80 °C and cryostatted with a microtome at 20 μm. According to the protocol of the fluorescent dye 4,6-diamidino-2-phenylindole (DAPI) nuclei staining process, desired tumor tissues were incubated with antigen presenting cell (APC) specific antibody anti-CD8 (Bio Legend) for the stain of CD8+ T-cells. Keyence fluorescence microscopy was used to visualize tumor tissues (BZ-9000, Tokyo, Japan). On a microscopic view, the nuclei staining was visualized as green, while the expression of CD8+ T-cells was visualized as red. The number of CD8+ T cells expressed in tumor microenvironment (TME) was calculated and optimized using ImageJ software.

S-1.8: Hematoxylin and Eosin (H&E) Staining for Organ Histopathological Study

We cleansed the mice's skin using hair removal cream to examine the in vivo biocompatibility of the tested sample on C57BL/6N mice. We used a home-made patch (1 cm × 1 cm; three layers of gauze; Hakujuji, Japan) and a tape (CATHEREEP FS dressing tape; Nichiban, Tokyo, Japan) to apply 200 μL of the tested solutions to the mice's dorsal portion for 24 h, with three consecutive doses at seven-days interval. Body weights were taken at one-day intervals. Mice organelles such as skin, liver, kidney, spleen, heart, and lung were collected after the third dose was administered for histological examination. The organelles were rinsed in PBS before being immersed in a 0.4 % formaldehyde solution for 6 h. They were then maintained at −80 °C and cryosectioned into 20 μm thickness with a microtome (CM1860UV; Leica Biosystems, Heidelberger, Nußloch, Germany), and arranged on a glass slide. Hematoxylin and eosin (H&E) solutions were used to stain the desired tissues, as described below. The desired tissues on the slide were cleaned with acetone and 99%, 90%, 70%, and 50% ethanolic aqueous solutions, then stained with hematoxylin solutions for 6-min before being rinsed with water to eliminate excess hematoxylin color. Stain for another 30 s with eosin solutions before washing with water to remove the color. Dry the tissues after washing them with a 25 percent, 50 percent, 75 percent, 90 percent, or 99 percent ethanolic aqueous solution. The tissues were adjusted on a glass slide with a mounting solution for microscopic entellanment before being used to evaluate the histopathological effect, using BZ-900 color microscopy (Keyence Co., Osaka, Japan).

S-2. Supportive Figures

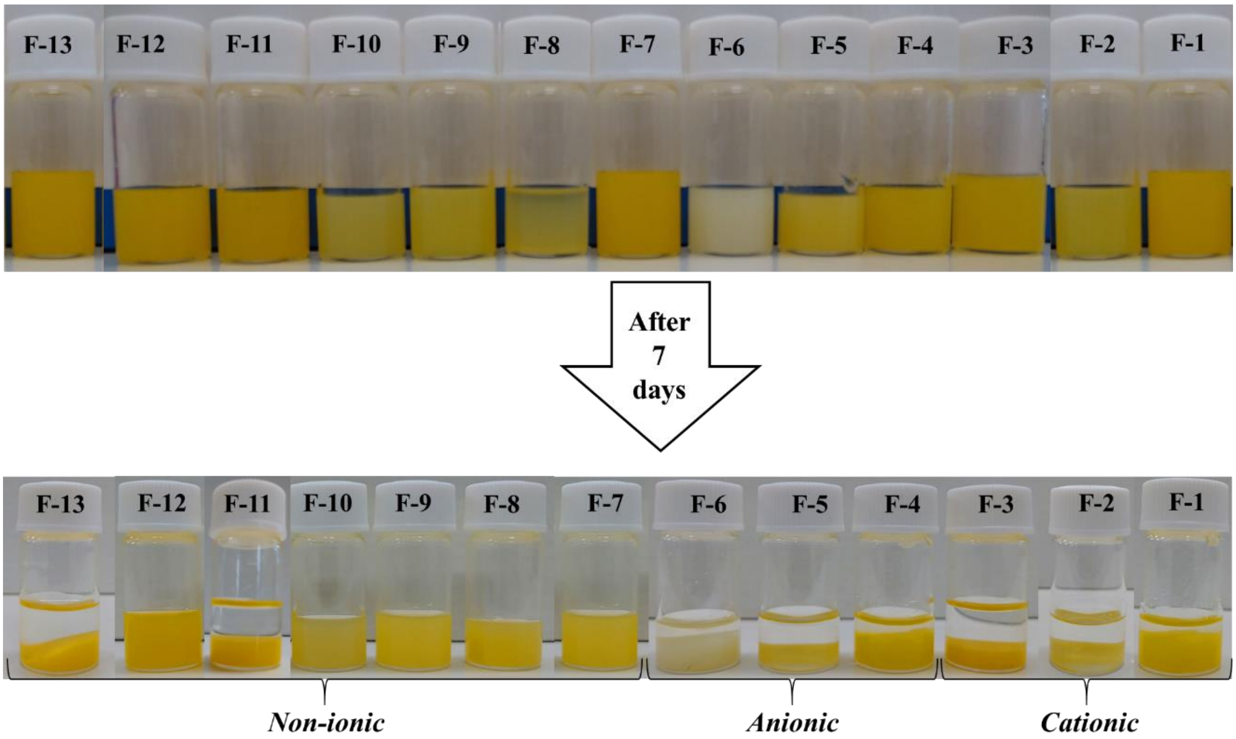

Figure S1. The physical stability of LCNDFs was observed by the visual observation. The upper image represented on the 0-day's formulations, and the lower image represented the 7th day's formulations. After a week, the unstable formulations were precipitated or separated into two phases. In 2nd image's, the upper, clear transparent phase representes the oil phase (IPM), and the lower precipitation phases contain FITC-OVA antigenic protein.

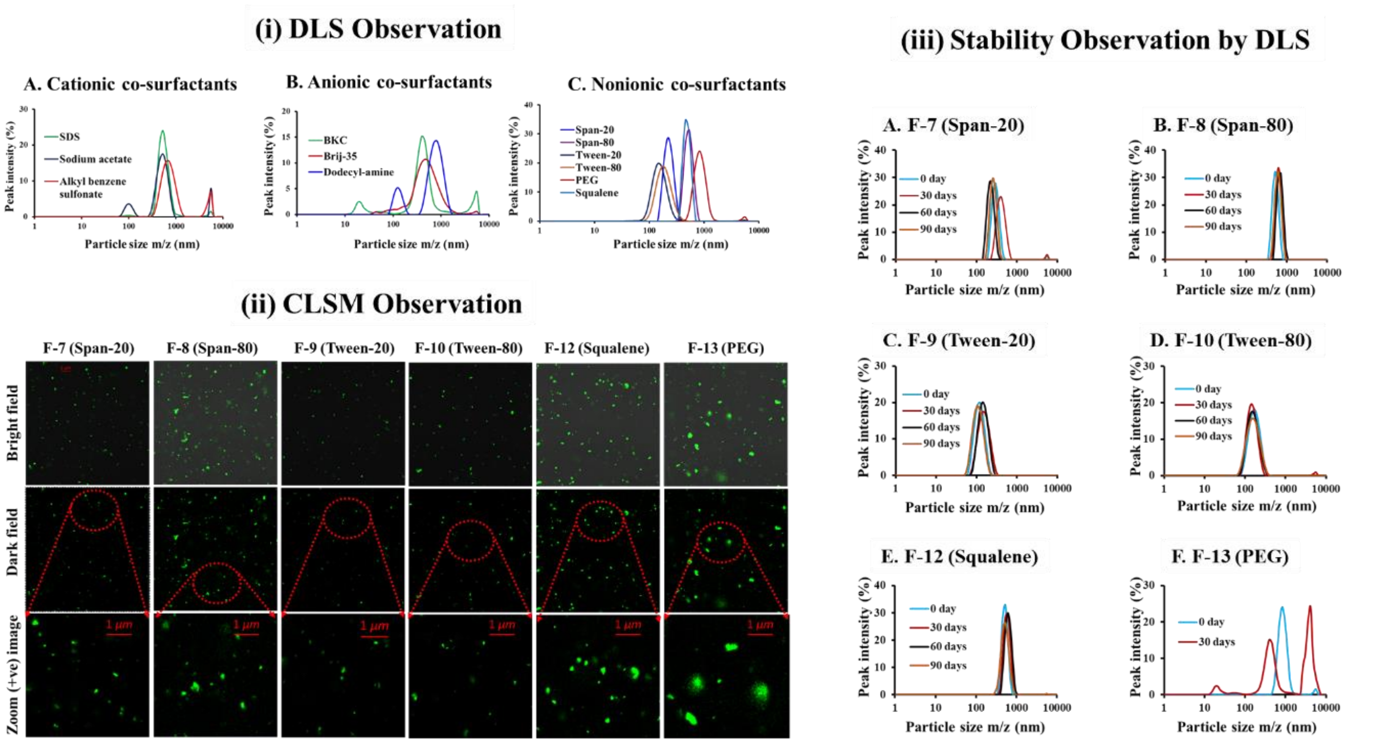

Figure S2. (i) DLS was used to investigate the particle size of LCNDFs. Average valued of 10 replicated measurements were represented as main particle size. (ii) CLSM detected FITC-OVA nanoparticles moving freely in IPM for stable formulations. (iii) DLS investigated the physical stability of LCNDFs by measuring particle size over three-month period. Average valued of 10 replicated measurements were represented as main particle size.

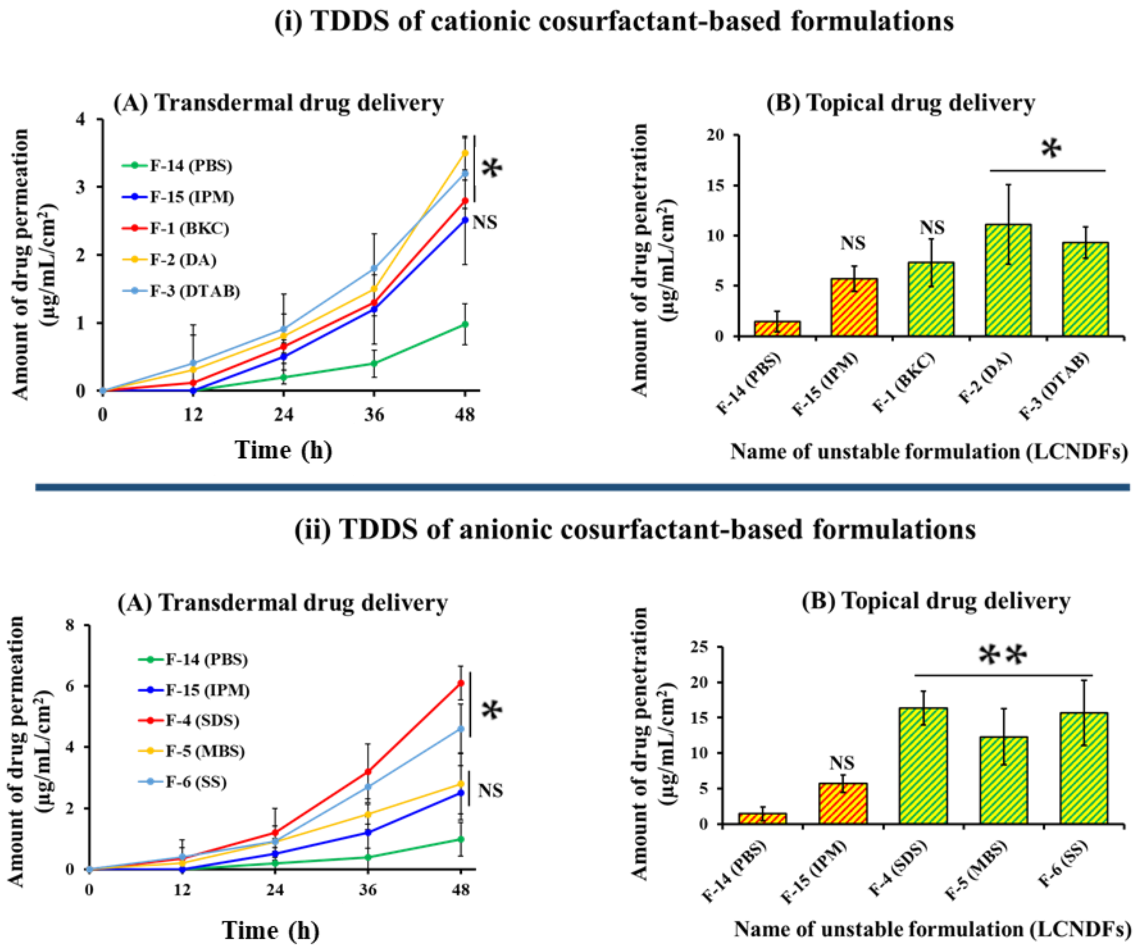

Figure S3. (i) Transdermal and topical drug delivery of cationic cosurfactant-based formulations were performed on YMPC-pig skin by using Franz diffusion cells for 48 h. (ii) Transdermal and topical drug delivery of anionic cosurfactant-based formulations were performed on YMPC-pig skin by using Franz diffusion cells until 48 h. Here, mean ± SD (n = 3), *p < 0.1, **p < 0.01, and NS = not significant.

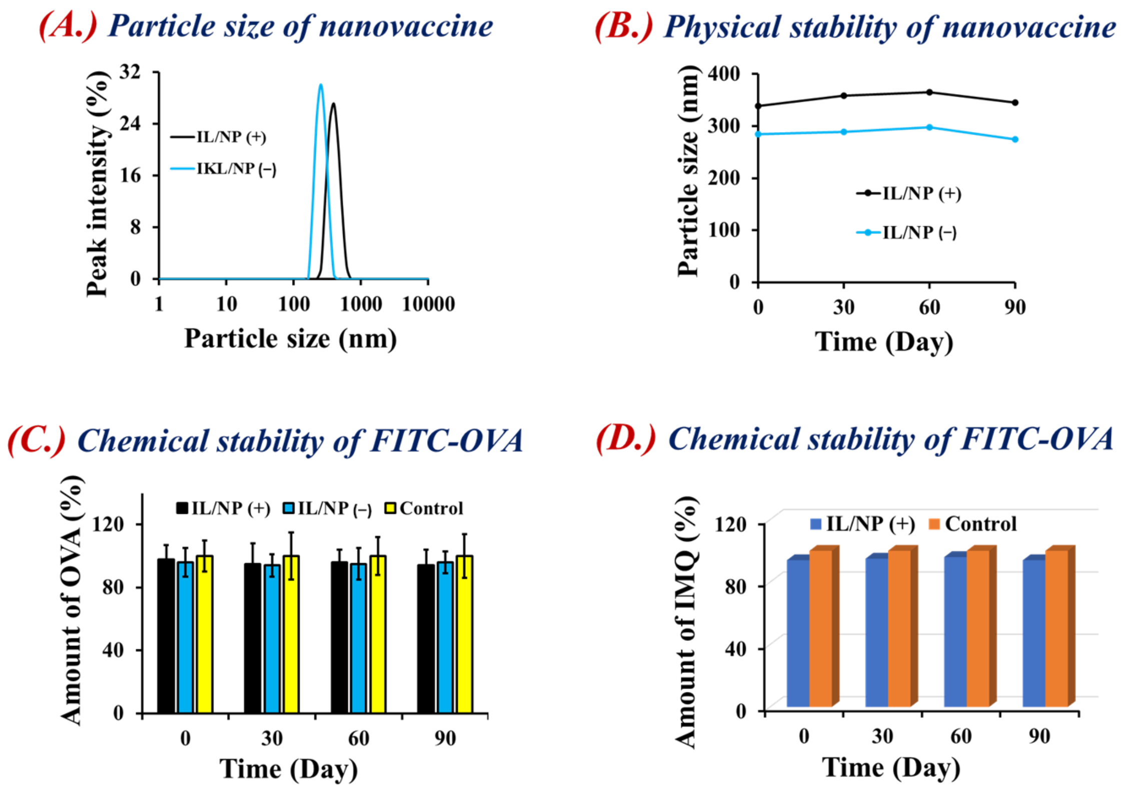

Figure S4. (A) Particle size of nanovaccine was determined by DLS. Average valued of 10 replicated measurements were represented as main particle size. (B) Particle size stability of nanovaccine up to three months. (C) Chemical stability of FITC-OVA in nanovaccine. (D) Chemical stability of IMQ in immunomodulator based nanovaccine.

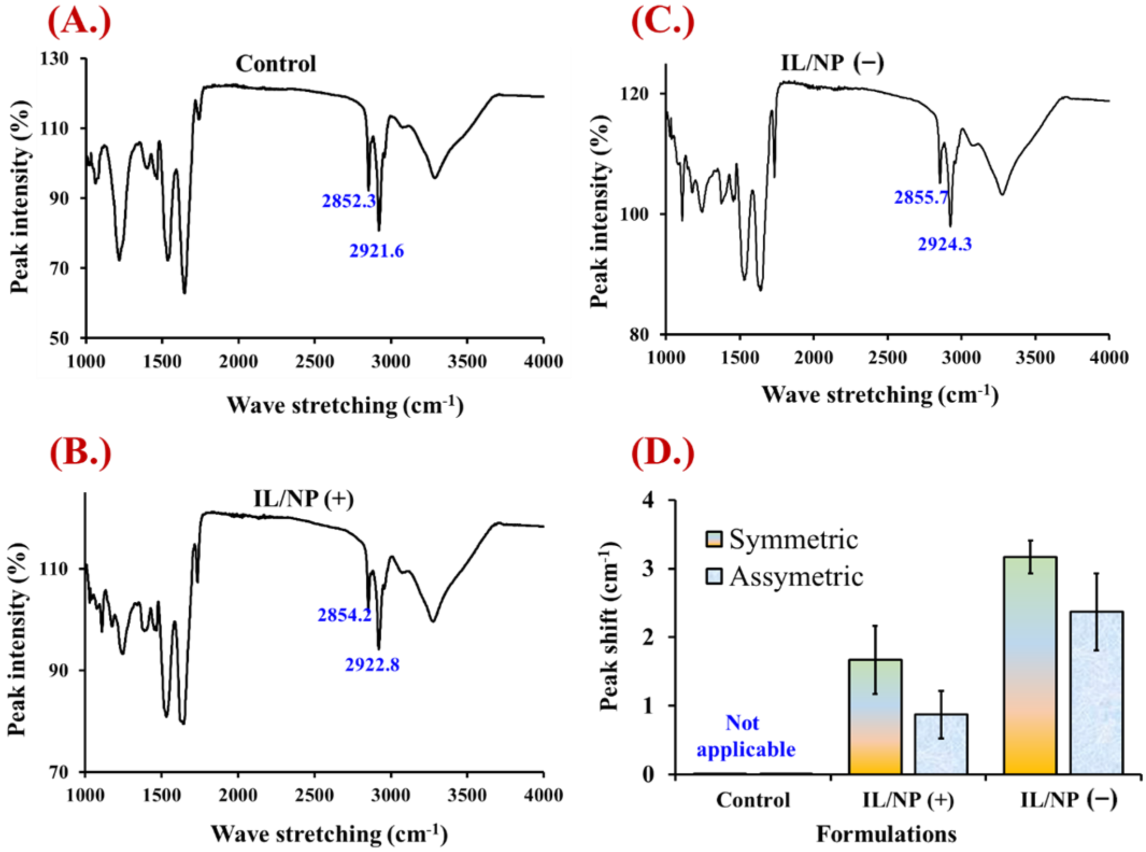

Figure S5. Effect of nanovaccine on YMP-skin's SC layer. (A) FTIR chromatograph of control group's mice skin; (B) FTIR chromatograph of IL/NP (+) group's mice skin; (C) FTIR chromatograph of IL/NP (−) group's mice skin; and (D) represented the statistical analysis of symmetric and asymmetric peak shift of different groups of drug formulation. The ability of drug delivery can be explained by a shift in the symmetric and asymmetric lipidic arrangement in the skin's structure. The more shift is allowed more amount of drug diffusion through the skin layer (epidermis or dermis, even in bleed circulation system).

S-3. Supportive Tables

Table S1. Maximum drug loading profile of LCNDFs.

Formulations Name	Symbol of the Formulations	Composition Names		Amount of OVA-Drug loading (mg/mL)
		Name of the Surfactants (2.5 wt.%)	Name of the Co-surfactants (2.5 wt.%)	
		Cationic cosurfactants		
L-BKC-NDF	F-1	[EDMPC][Lin]	BKC	0.89 ± 0.35
L-DA-NDF	F-2	[EDMPC][Lin]	DA	1.02 ± 0.65
L-DTAB-NDF	F-3	[EDMPC][Lin]	DTAB	0.98 ± 0.56
		Anionic cosurfactants		
L-SDS-NDF	F-4	[EDMPC][Lin]	SDS	3.02 ± 0.89
L-MBS-NDF	F-5	[EDMPC][Lin]	MBS	1.41 ± 0.35
L-SS-NDF	F-6	[EDMPC][Lin]	SS	1.22 ± 0.68
		Non-ionic or Neutral cosurfactants		
L-S-20-NDF	F-7	[EDMPC][Lin]	Span-20	7.11 ± 1.20
L-S-80-NDF	F-8	[EDMPC][Lin]	Span-80	6.49 ± 0.95
L-T-20-NDF	F-9	[EDMPC][Lin]	Tween-20	7.18 ± 0.58
L-T-80-NDF	F-10	[EDMPC][Lin]	Tween-80	6.63 ± 0.85
L-Brij-NDF	F-11	[EDMPC][Lin]	Brij-35	0.89 ± 0.45
L-Sq-NDF	F-12	[EDMPC][Lin]	Squalene	6.31 ± 0.99
L-PEG-NDF	F-13	[EDMPC][Lin]	PEG	3.33 ± 0.51
		Control group or without surfactant		
Oil NDF	F-15	-	-	0.78 ± 0.36

Here, the measurement of drug amount was considered to be the average values of two samples measurements. Mean values as n = 3 ± SD.

Table S2. In vitro transdermal and topical drug delivery of cationic and anionic cosurfactant-based formulations.

**Formulations name**	**Transdermal drug delivery or Permeation of the drug (μg/mL/cm²)**					**Topical delivery or Penetration (μg/mL/cm²)**
	Time (h)					Extraction
	0	12	24	36	48	
F-14 (PBS)	0	0	0.2	0.4	0.98	3.49
F-15 (IPM)	0	0	0.5	1.2	2.51	5.26
Cationic cosurfactants- based formulation						
F-1 (BKC)	0	0.12	0.65	1.3	2.8	7.31
F-2 (DA)	0	0.31	0.81	1.5	3.5	11.12
F-3 (DTAB)	0	0.41	0.91	1.8	3.2	9.3
Anionic cosurfactants- based formulation						
F-4 (SDS)	0	0.35	1.2	3.2	6.1	16.35
F-5 (MBS)	0	0.2	0.9	1.8	2.8	12.3
F-6 (SS)	0	0.41	0.91	2.7	4.6	15.68

Here, the measurement of drug amount was considered to be the average values of three samples measurements. Mean values ± SD (n = 3).

Table S3. In vitro transdermal and topical drug delivery of nonionic cosurfactant based formulations.

**Formulations name**	**Transdermal drug delivery or Permeation of the drug (μg/mL/cm²)**					**Topical delivery or Penetration (μg/mL/cm²)**
	Time (h)					Extraction
	0	12	24	36	48	
F-14 (PBS)	0.00	0.00	0.21	0.41	0.98	3.49
F-15 (IPM)	0.00	0.00	0.50	1.22	2.51	5.26
F-7 (Span-20)	0.00	0.95	2.59	5.12	8.54	37.49
F-8 (Span-80)	0.00	0.81	1.98	4.58	7.79	32.10
F-9 (Tween-20)	0.00	0.93	2.22	4.31	7.54	33.21
F-10 (Tween-80)	0.00	0.65	1.75	4.08	6.65	30.25
F-11 (Brij-35)	0.00	0.00	0.40	1.52	2.65	7.65
F-12 (Squalene)	0.00	0.41	1.12	3.23	6.30	30.21
F-13 (PEG)	0.00	0.75	1.51	4.11	7.71	31.21

Here, the measurement of drug amount was considered to be the average values of three samples measurements. Mean values ± SD (n = 3).
